# Compressed Adaptive-Sampling-Rate Image Sensing Based on Overcomplete Dictionary

**DOI:** 10.3390/e27070709

**Published:** 2025-06-30

**Authors:** Jianming Wang, Dingpeng Li, Qingqing Yang, Yi Peng

**Affiliations:** School of Information Engineering and Automation, Kunming University of Science and Technology, Kunming 650500, China; 20220079@kust.edu.cn (J.W.); lidingpeng@stu.kust.edu.cn (D.L.); 20090119@kust.edu.cn (Q.Y.)

**Keywords:** compressed sensing, overcomplete ridgelet dictionary, adaptive sampling rate, wireless sensor image capture networks

## Abstract

In this paper, a compressed adaptive image-sensing method based on an overcomplete ridgelet dictionary is proposed. Some low-complexity operations are designed to distinguish between smooth blocks and texture blocks in the compressed domain, and adaptive sampling is performed by assigning different sampling rates to different types of blocks. The efficient, sparse representation of images is achieved by using an overcomplete ridgelet dictionary; at the same time, a reasonable dictionary-partitioning method is designed, which effectively reduces the number of candidate dictionary atoms and greatly improves the speed of classification. Unlike existing methods, the proposed method does not rely on the original signal, and computation is simple, making it particularly suitable for scenarios where a device’s computing power is limited. At the same time, the proposed method can accurately identify smooth image blocks and more reasonably allocate sampling rates to obtain a reconstructed image with better quality. The experimental results show that our method’s image reconstruction quality is superior to that of existing ARCS methods and still maintains low computational complexity.

## 1. Introduction

### 1.1. Motivation

In recent years, wireless image sensors have shown potential for application in monitoring, tracking, and other fields owing to their low cost and convenience. However, in these resource-limited sensors, traditional image sampling and encoding methods are too complex, so the compressed sensing (CS) method is considered more suitable. First, the CS sampling process is a linear dimension-reducing process; the sampling and compression operations are completed simultaneously, and the calculations are simple. Second, CS can sample at a rate much lower than the Nyquist sampling rate by utilizing the sparsity of the signal, which further reduces the performance requirements for sampling equipment. Finally, the complex calculation is moved to the reconstruction end, which is also consistent with the structural characteristics of a distributed system. These advantages have attracted the attention of researchers, and a series of methods based on CS have been proposed [1,2].

However, there are still some problems that require further research when applying the CS method to distributed systems, one of which is how to implement the adaptive rate compression sensing (ARCS) method [3]. The CS method reduces the dimensions of the original signal in the sampling process and obtains the CS measurement directly. In terms of operation complexity reduction, this is an advantage; however, it also means that the original signal is hidden in the CS measurement and is unknown to the sampling device, which results in new problems, including the ARCS problem.

Different parts of a natural image have different complexities. Traditional image encoding techniques divide an image into multiple blocks and allocate different encoding rates based on the complexity of the blocks. However, in CS, since the original signal is unknown, it is difficult to estimate the complexity of a block and assign an appropriate sampling rate for it. This is the ARCS problem.

### 1.2. Related Works

Many researchers have conducted extensive studies to achieve ARCS. In [4,5,6], the authors assumed that the original signal was fully measured; then, an appropriate sampling rate was set, and the reconstruction quality was improved. However, the full measurements increases the sampling pressure of the sensor, resulting in increased costs and energy consumption, which does not fully utilize the advantages of CS. Yu et al. [7] proposed a solution that obtains low-resolution images through an auxiliary sensor and extracts salient features from the low-resolution image. However, although this method reduces the sampling pressure to some extent, the problem is not fundamentally solved.

To achieve ARCS without relying on the original image, Li et al. [8] proposed a method based on sensed entropy. The sampling rate of the image block is allocated based on the sensed entropy, which can be obtained by using CS measurement. A saliency-based [9] ARCS method is proposed in [10]. In this method, saliency is used to characterize the differences between blocks, while the differences are used as the basis for sampling-rate allocation. However, misclassification sometimes occurs with the above two methods, leading to block effects in the reconstructed images. To address the block effect problem, a method based on empirical modal decomposition (EMD) is proposed in [11]. EMD is used to obtain an energy distribution map of the high-frequency components, and adaptive sampling rate allocation is realized based on the energy distribution map. The method improves reconstruction quality; however, the calculation complexity of the EMD process is relatively high. In [12], an ARCS method based on statistical characteristic estimation and signal prior probability is proposed. This method is computationally simple but requires an understanding of the prior probability distribution. In [13,14], the authors proposed two adaptive rate video CS methods based on motion estimation. Adaptive rate allocation was achieved by estimating the motion of the foreground objects in the video. However, these methods assume a strong correlation between blocks and are therefore not applicable to image signals.

Although the previous studies have proposed diverse adaptive sampling methods, these approaches still suffer from several limitations, including dependence on the original image, high computational complexity, and low reconstruction quality. To provide a comprehensive understanding, Table 1 compares the strengths and weaknesses of these methods.

### 1.3. Proposed Work

To address the limitations of the previous methods and improve the performance of distributed systems based on a CS scheme, a new adaptive-rate compression block sensing method based on an overcomplete ridgelet dictionary (ABCS-RDET) is proposed. This method pays special attention to the following aspects during design:

First, the proposed method depends only on the compressed domain signal, and the original signal can be unknown.

Second, it uses an overcomplete ridgelet dictionary to sparsely represent the signal. Due to its ability to accurately capture the ridge features in natural images, especially the edge parts, the overcomplete ridgelet dictionary has good sparsity performance on natural images. In this method, the characteristics of the dictionary are utilized to achieve an efficient sparse representation of the signals.

Third, when using an overcomplete dictionary, the excessive number of atoms in the dictionary can have a negative impact on the speed of atomic matching. A dictionary partitioning method was designed to solve this problem. By dividing the overcomplete dictionary into smooth parts and texture parts, the number of candidate atoms in the matching process is reduced, and the matching speed is improved. Thus, atomic matching calculation can be used on a resource-limited device, and the matching results can be used to distinguish between smooth blocks and texture blocks.

Finally, based on the proportion of smooth blocks in an image, the overall complexity of the image can be estimated. Depending on the complexity estimation, different rate allocation strategies can be used to the allocate appropriate sampling rates for each block, and adaptive rate sampling can be achieved.

Based on the above ideas, the following features are ultimately achieved: (a) the method does not rely on the original signal, (b) its computational complexity is very low, (c) it improves the quality of the reconstructed image. These features ultimately make this method suitable for practical applications, which demonstrates good performance.

Finally, from a technical perspective, the two specific contributions of this method are as follows:

1. An adaptive residual energy hard-threshold classification method based on an overcomplete dictionary is proposed. This method is independent of the original signal as well as its prior statistics and has a simple implementation process and a fast calculation speed.
2. We developed a dictionary partition method. By partitioning the dictionary, the method significantly boosts both the speed and the accuracy of image block classification, thereby enhancing the image reconstruction quality.

The rest of this article is organized as follows: Section 2 briefly introduces the methodological background; Section 3 provides a specific introduction to the proposed method; Section 4 provides the experimental results and the corresponding analysis; Section 5 is the summary.

## 2. Methodological Background

### 2.1. Block Compressed Sensing

The sampling process of CS can be expressed as

(1) y=Φx,

where $x\in R^{n}$ is the original signal, $\Phi \in R^{m\times n}(m<n)$ is the measurement matrix, and $y\in R^{m}$ is the CS measurement. For signal x, the CS method requires it to be “sparse”; that is, most of its elements are zero, and only a small number of elements have significant non-zero values. If the number of non-zero elements is k, signal x is said to be “k-sparse”. Natural image signals can be represented as sparse coefficients by using certain sparse dictionaries, so natural images can also be sampled and reconstructed using CS.

However, since the total number of pixels n is very large for a whole image, the memory consume for related Φ becomes unacceptable. In order to solve this problem, block compressed sensing (BCS) [15] was proposed. Assuming that the image collected by the current sensor is X, X can be decomposed into I non-overlapping sub-blocks, and the size of each block is B×B. Then, each sub-block can be vectorized as $x_{i}\in R^{n}$, where $n=B^{2}$, and i is the image block index, i∈1,2,3,…,I. Each $x_{i}$ can be separately sampled using a much smaller matrix $\Phi_{b}\in R^{m\times n}$, and the block measurement $y_{i}$ can be obtained from

(2) $$y_{i}=\Phi_{b}x_{i}=\Phi_{b}\Psi a_{i},$$

where Ψ is the sparse basis, and $a_{i}$ is the sparse coefficient of $x_{i}$.

### 2.2. Compressed Sensing Based on Overcomplete Dictionary

In the CS method, the sparse representation of signals is the most important prerequisite, and signals should be represented as sparsely as possible. For the effective representation of images, the images must be localized as well as oriented and have a suitable bandpass [16,17]. The overcomplete dictionary is one of the representations that has these properties [18]. Compared with orthogonal sparse bases, overcomplete dictionaries tend to have stronger sparse representation capabilities [19]. Therefore, the CS method based on an overcomplete dictionary has been of interest to researchers.

An overcomplete dictionary can be represented as a two-dimensional matrix $D\in R^{n\times C}$, where each column vector is called a dictionary atom, which is vectorized from a two-dimensional dictionary atom matrix. The number of atoms C is often much greater than n. In an overcomplete dictionary, the signal can be expressed as

(3) $$x_{i}=Da_{i}=\sum_{j=1}^{k}a_{j}d_{j},$$

where $a_{i}$ is a sparse vector, k is the number of non-zero elements in $a_{i}$, $a_{j}$ is the value of the j-th non-zero element, and $d_{j}$ is the atom in D corresponding to the position of $a_{j}$. Now signal $x_{i}$ is represented as a k-sparse signal under dictionary D, and

(4) $$y_{i}=\Phi_{b}x_{i}=\Phi_{b}Da_{i}=A_{b}a_{i},$$

where $A_{b}=\Phi_{b}D$ is a compressed dictionary.

If $x_{i}$ is to be accurately reconstructed from $y_{i}$, the measurement matrix $\Phi_{b}$ must meet the D-RIP [20,21] characteristics,

(5) $$\left(1-\delta_{k}\right){\left\|v\right\|}_{2}^{2}\leq {\left\|\Phi_{b}v\right\|}_{2}^{2}\leq \left(1+\delta_{k}\right){\left\|v\right\|}_{2,}^{2}$$

where $\delta_{k}\in \left(0,1\right)$ is a constant, and v is any vector that concentrates in the subspace formed by all k column subsets of D.

E. J. Candès [21] proved that both the Gaussian random matrix [22] and the Bernoulli random matrix [23] can satisfy the requirements of D-RIP. In this paper, a random Gaussian matrix is used as the measurement matrix. In [21], it is also proven that the measurement number m and the sparsity k have the following relationship:

(6) $$m\geq k\cdot log\left(C/k\right).$$

Equation (6) shows that the sparsity k determines the number of measurements m. Therefore, setting the measurement number according to the sparseness can effectively save resources.

An approximate ${\tilde{a}}_{i}$ of solution $a_{i}$ can be obtained by solving the following model [20,21]:

(7) $$\underset{{\tilde{a}}_{i}}{{\tilde{a}}_{i}=min{\left\|{\tilde{a}}_{i}\right\|}_{1}}\;such\;that\;{\left\|A{\tilde{a}}_{i}-y_{i}\right\|}_{2}\leq \epsilon ,$$

where ϵ is the upper boundary of the signal noise. Many recovery algorithms can be used to solve (7), the popular algorithms for which are BP [24], GPSR [25], etc.

### 2.3. Discrete Overcomplete Ridgelet Dictionary

An overcomplete ridgelet dictionary is a commonly used overcomplete dictionary. It can efficiently capture the texture structure and edge information of an image and has high-quality sparse representation ability [26]. The specific discrete ridgelet dictionary [27] described below is used in the proposed method.

The basis in the dictionary is two-dimensional, noted as $d_{c}(z)\in R^{B\times B}$, which is generated by the parameter space $\Gamma_{\gamma }=\left\{\left(\theta ,s,t\right)|\theta \in \left[0,\pi \right],s\in \left[0,3\right],t\in \Gamma_{t}\right\}$, where θ is the direction parameter, and s is the scale parameter. The value range of parameter t is related to the direction parameter θ:

(8) $$\Gamma_{t}=\left\{\begin{array}{l} \left[0,B\left(sin\theta +cos\theta \right)\right],\;\;\;\theta \in \left[0,\frac{\pi }{2}\right]\\ \left[Bcos\theta ,Bsin\theta \right],\;\;\;other \end{array}\right.,$$

$d_{c}(z)$ is generated by the above parameters as

(9) $$d_{c}\left(z\right)=\frac{1}{w}\left[e^{\frac{-(s_{c}r_{c}z^{T}-t_{c}{)}^{2}}{2}}-\frac{1}{2}e^{\frac{-(s_{c}r_{c}z^{T}-t_{c}{)}^{2}}{8}}\right],$$

where $z=z\left(z_{x},z_{y}\right)\in {\left[0,1,2,\ldots ,B-1\right]}^{2}$ is an atomic position variable, $r_{c}=\left(cos\theta_{c},sin\theta_{c}\right)$ is a rotation vector determined by parameter θ, and w is the weight coefficient used to normalize the atoms.

To obtain dictionary atoms, the above continuous function should be discretized. In this method, the direction parameter θ is sampled at intervals of π/36; the value of the scale parameter s is 0.2$k_{s}$, $k_{s}=0,1,2,\ldots ,15$; the sampling interval of the shift parameter t is taken as $2-0.1k_{s}$. And it is necessary to set the atom energy threshold $\tau_{D}$ to filter low-energy atoms; it is set as $\tau_{D}=2.5$ here, and the ridgelet dictionary has a total atom number C=2806.

To sparsely represent the vectorized signal, the two-dimensional atom $d_{c}(z)$ is vectorized as $d_{c}\in R^{n}$, c=1,2,3,…,C, which finally generates the 2-D dictionary $D=\left(d_{1},d_{2},d_{3},\ldots {,d}_{C}\right)$.

## 3. Adaptive Rate Method

### 3.1. Identification of Smooth Blocks and Texture Blocks

Equation (3) shows that signal $x_{i}$ can be represented by dictionary D, and sparse coefficients $a_{i}$, and $a_{i}$ can be obtained by solving the following model [19]:

(10) $$a_{i}=min{\left\|a_{i}\right\|}_{1}\;such\;that\;x_{i}=Da_{i}.$$

A suitable dictionary D can often make most elements in $a_{i}$ approach 0, while only k elements have significantly larger values. At this time, $a_{i}$ is a compressible signal, and the original signal $x_{i}$ can be represented by k large values in $a_{i}$ [28]:

(11) $$x_{i}=\sum_{j=1}^{k}a_{j}d_{j}+\varepsilon_{i},$$

where $\varepsilon_{i}$ is the residual.

**Figure 1:**
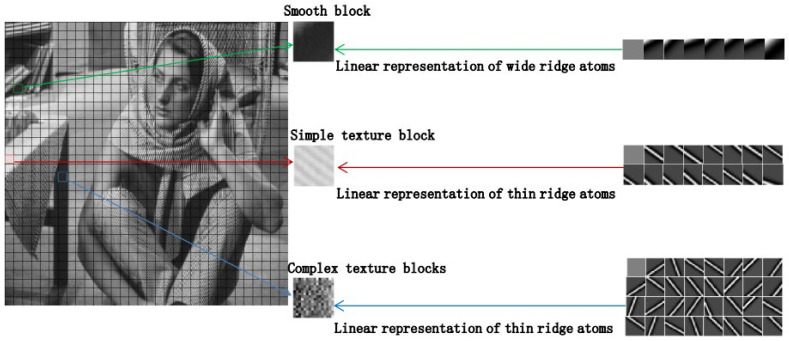
An example of atom matching.

The similarity between a dictionary atom and an image block is a crucial factor in determining atom matching. The higher the similarity between the dictionary atom and the image block, the more likely the atom is to be selected. For smooth image blocks, their similarity is higher with broad-ridge atoms. Therefore, wide ridge atoms are more likely to be selected to match smooth blocks. Further analysis showed that for a smooth image block, since the energy of the high-frequency components is very small, the linear combination of k wide ridge atoms can limit the error energy ${\left\|\varepsilon_{i}\right\|}_{2}^{2}$ to a very small range, while for a texture block, to limit ${\left\|\varepsilon_{i}\right\|}_{2}^{2}$ to a small range, more atoms with narrower ridges are needed. An example is shown in Figure 1.

Since the approximate representation of a smooth block requires only k atoms with wide ridges, the ridgelet dictionary $D\in R^{n\times C}$ can be decomposed into a smooth dictionary and a texture dictionary according to the ridges’ scale. The smooth dictionary is noted as $D_{s}\in R^{n\times C_{1}}$, which consists of 319 broad ridge atoms whose scale parameter $s\in \left[0,0.4\right]$,

(12) $$D_{s}≜\left\{{ds}_{c_{1}}|0\leq s\leq 0.4,{ds}_{c_{1}}\in D\right\}.$$

At the same time, the complement of the smooth dictionary about D is called the texture dictionary, noted as $D_{t}\in R^{n\times C_{2}}$, which consists of 2487 fine ridge atoms whose scale parameter $s\in \left(\left.0.4,3\right]\right.$,

(13) $$D_{t}≜\left\{{dt}_{c_{2}}|0.4<s\leq 3,{dt}_{c_{2}}\in D\right\}.$$

**Figure 2:**
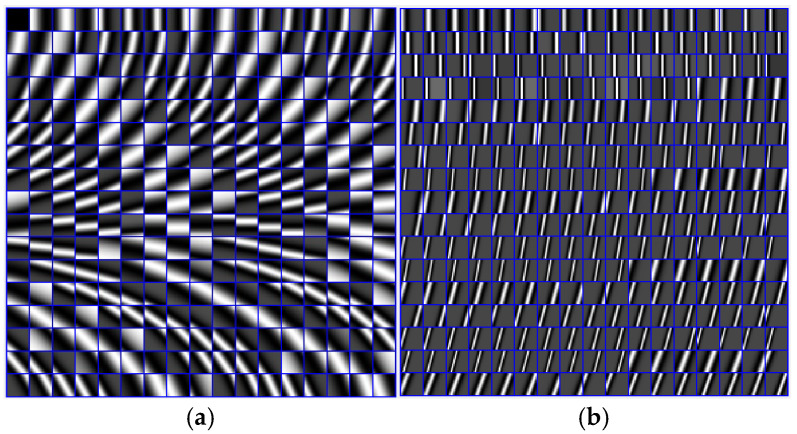
(**a**) Examples of the atoms in the smooth dictionary. (**b**) Examples of the atoms in the texture dictionary.

$C_{1}$ and $C_{2}$ are their atom numbers, ${C_{1}+C}_{2}=C$. Examples of atoms are shown in Figure 2.

By decomposing the dictionary D into $D_{s}$ and $D_{t}$, smooth blocks and texture blocks can quickly be distinguished: for an image block $x_{i}$ and a threshold τ, if there are k atoms in $D_{s}$ to control ${\left\|\varepsilon_{i}\right\|}_{2}^{2}\in \left[0,\tau \right]$, $x_{i}$ is considered as a smooth block; otherwise, it is a texture block. Since the size of $D_{s}$ is greatly smaller than that of D, the distinguishing process using $D_{s}$ only is much faster.

### 3.2. Block Classification in Compressed Domain

The block identification method proposed in Section 3.1 can be easily applied in the compressed domain. Using (11), $y_{i}$ can be expressed as

(14) $$y_{i}=\Phi_{b}x_{i}=\Phi_{b}\left(\sum_{j=1}^{k}a_{j}d_{j}+\varepsilon_{i}\right),$$

Noting that $\Phi_{b}\varepsilon_{i}=\omega_{i}$, $D_{sub}=\left[d_{1},d_{2}\ldots d_{k}\right]$, $\alpha_{i}={\left[a_{1},a_{2}\ldots a_{k}\right]}^{T}$, (14) can be rewritten as

(15) $$\omega_{i}=y_{i}-A_{sub}\cdot \alpha_{i},$$

where $A_{sub}=\Phi_{b}D_{sub}$, and $\omega_{i}$ is the residual.

The compressed dictionary $A_{b}=\Phi_{b}D$ can also be divided into two: the smooth compressed dictionary $A_{s}$ and the texture compressed dictionary $A_{t}$,

(16) $$A_{s}=\Phi_{b}D_{s}=\left[{as}_{1},{as}_{2},\ldots {,as}_{C_{1}}\right],$$

(17) $$A_{t}=\Phi_{b}D_{t}=\left[{at}_{1},{at}_{2},\ldots ,{at}_{C_{2}}\right].$$

If there are k atoms in $A_{s}$ to control $e_{i}={\left\|\omega_{i}\right\|}_{2}^{2}$ within a certain range, then it can be considered that the corresponding $x_{i}$ is a smoothing block.

Given $y_{i}$, $A_{s}$, and the allowed atom number ${k=K}_{s}$, the orthogonal matching pursuit (OMP) algorithm [29] can be used to determine $A_{sub}$ and $\alpha_{i}$.

First, the residual is initialized as $\omega_{i}^{0}=y_{i}$, the selected atomic index is initialized as $\Lambda_{i}^{0}=\emptyset$, and the selected atomic set is initialized as ${\tilde{A}}_{i}^{0}=\emptyset$. In each iteration, the algorithm matches and selects an atom from $A_{s}$ that is the most similar to the current residual. That is, in the t-th iteration, it finds the index ${\tilde{c_{1}}}_{i}^{t}$ corresponding to

(18) $${\tilde{c_{1}}}_{i}^{t}={argmax}_{c_{1}=1,\ldots ,C_{1}}\left|\langle \omega_{i}^{t-1},{as}_{c_{1}}\rangle \right|,$$

where the symbol $\left|\langle \cdot ,\cdot \rangle \right|$ represents the absolute value of the inner product, and ${as}_{c_{1}}$ is an atom of $A_{s}$. Next, $\Lambda_{i}^{t}$ and ${\tilde{A}}_{i}^{t}$ can be updated:

(19) $$\Lambda_{i}^{t}=\Lambda_{i}^{t-1}\cup {\tilde{c_{1}}}_{i}^{t},$$

(20) $${\tilde{A}}_{i}^{t}=\left[{\tilde{A}}_{i}^{t-1},{as}_{{\tilde{c_{1}}}_{i}^{t}}\right].$$

Then, the linear representation coefficient ${\tilde{\alpha }}_{i}^{t}$ can be solved using the least squares method:

(21) $${\tilde{\alpha }}_{i}^{t}={\left({{\tilde{A}}_{i}^{t}}^{T}\cdot {\tilde{A}}_{i}^{t}\right)}^{-1}\cdot {{\tilde{A}}_{i}^{t}}^{T}\cdot y_{i}.$$

Finally, the residual $\omega_{i}^{t}$ can be updated:

(22) $$\omega_{i}^{t}=y_{i}-{\tilde{A}}_{i}^{t}\cdot {\tilde{\alpha }}_{i}^{t}.$$

When $t=K_{s}$, the iteration stops, and $e_{i}={\left\|\omega_{i}^{t}\right\|}_{2}^{2}$ is output.

**Figure 3:**
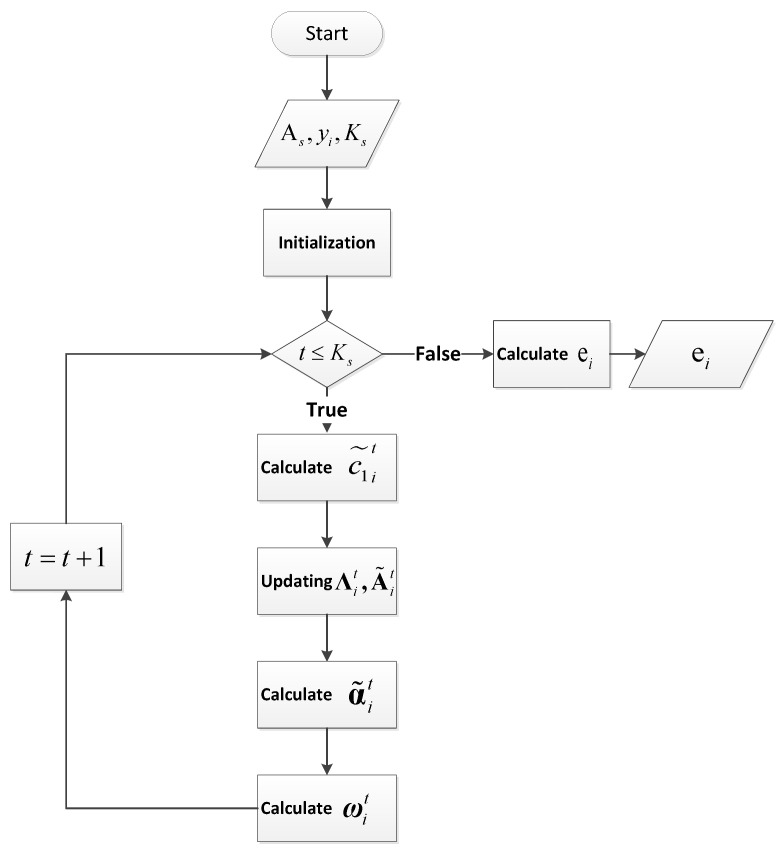
Flowchart of algorithm.

The algorithm details are shown in Algorithm 1 and Figure 3.
**Algorithm 1** Classification algorithm**Input:** $A_{s}$: smooth compressed dictionary. $y_{i}$: measurement value of image $x_{i}$ $K_{s}$: the number of iterations of the algorithm. **Initialization:** $\omega_{i}^{0}=y_{i},{\tilde{A}}_{i}^{0}=\emptyset \;,\Lambda_{i}^{0}=\emptyset$ **while** $t\leq K_{s}$ **do** ${\tilde{c_{1}}}_{i}^{t}={argmax}_{c_{1}=1,\ldots ,C_{1}}\left|\langle \omega_{i}^{t-1},{as}_{c_{1}}\rangle \right|$; $\Lambda_{i}^{t}=\Lambda_{i}^{t-1}\cup {\tilde{c_{1}}}_{i}^{t}$; ${\tilde{A}}_{i}^{t}=\left[{\tilde{A}}_{i}^{t-1},{as}_{{\tilde{c_{1}}}_{i}^{t}}\right]$; ${\tilde{\alpha }}_{i}^{t}={\left({{\tilde{A}}_{i}^{t}}^{T}\cdot {\tilde{A}}_{i}^{t}\right)}^{-1}\cdot {{\tilde{A}}_{i}^{t}}^{T}\cdot y_{i}$; $\omega_{i}^{t}=y_{i}-{\tilde{A}}_{i}^{t}\cdot {\tilde{\alpha }}_{i}^{t};t=t+1$ **end** $e_{i}={\left\|\omega_{i}^{t}\right\|}_{2}^{2}$ **Output:** $e_{i}$: the residual energy of the i-th block’s image

The CS measurement results of the entire picture are stored in a matrix $Y=\left[y_{1},y_{2},\ldots ,y_{I}\right]$. Using the above method, the residual energy vector $e=\left(e_{1},e_{2},\ldots ,e_{I}\right)$ corresponding to all blocks can be obtained. Then e is normalized as $\bar{e}$ using the Min–Max method [30],

(23) $$\bar{e}=\frac{e-\min\left(e\right)}{\max\left(e\right)-\min\left(e\right)},$$

where min(e) represents the minimum value of the element in vector e, and max(e) represents the maximum value of the element in vector e. Finally, a fixed threshold $\tau_{s}$ can be set to classify the smooth and textured blocks.

Dictionary $A_{b}$ is decomposed as $A_{s}$ and $A_{t}$, and the matching operation uses $A_{s}$ only, whose size is much smaller than that of $A_{b}$; thus, the classification operation is simple and fast. However, we should also point out that for the classified texture blocks, they can be further distinguished as simple texture blocks and complex texture blocks using similar methods, if necessary. The differences are that k should be set to a larger value and the candidate dictionary becomes $A_{t}$**.** Such differences lead to a relatively complex computation.

### 3.3. Adaptive Rate Allocation

We assume that the total sampling rate r of an image is fixed, and the main purpose of the proposed method is to reasonably allocate the total number of samples S to each block, where S=r·n·I.

First, before starting the adaptive rate allocation, in order to obtain the initial CS measurements, a fixed low-rate sampling matrix $\Phi_{l}\in R^{m_{l}\times n}$ should be used for quick measurement, and the corresponding $y_{i}^{l}\in R^{m_{l}}$ can be obtained, where $m_{l}$ is the size and is a small number.

Second, using $y_{i}^{l}$ and the proposed method in Section 3.2, the image blocks can be divided into smooth blocks (note das $C_{s}$ class) and texture blocks (noted as $C_{t}$ class).

Third, in order to utilize the limited sampling number more effectively, a dynamic rate allocation strategy based on the complexity of the entire image is proposed. The proportion of smooth blocks in an entire image can be used to characterize its overall complexity. Note that the number of $C_{s}$ blocks is $N_{s}$; when $N_{s}\geq I/2$, the entire image is considered a simple one; otherwise, it is considered a complex one. For a simple image, it is reasonable that the total sampling number is sufficient, since smoothing blocks require a few sampling resources, leaving enough remaining resources for complex blocks. For complex images, the situation is the opposite: a more accurate allocating method is required.

When there is a complex image, its texture blocks should be further divided into simple texture blocks (note as $C_{t_{1}}$ class) and complex texture blocks (note as $C_{t_{2}}$ class). The specific dividing method is described in the last part of Section 3.2, where $A_{t}$ is selected as the candidate atom set, $k=K_{t}$, and a new threshold $\tau_{t}$ needs to be set.

For simpler images whose blocks are divided into two categories, $C_{s}$ and $C_{t}$, a fixed initial sampling number $m_{l}$ is assigned to $C_{s}$ blocks; that is, $m_{C_{s}}=m_{l}$, where $m_{C_{s}}$ is the sampling number of $C_{s}$, and then the sampling number of the $C_{t}$ blocks can be decided,

(24) $$m_{C_{t}}=round\left(\frac{S-m_{C_{s}}\cdot N_{s}}{N_{t}}\right),$$

where round() represents the rounding function, and $N_{t}$ is total number of $C_{t}$ blocks. Then, it is necessary to check if $m_{C_{t}}$ is greater than a given maximum number $m_{C_{t}}^{ul}$, which should be set as $m_{C_{t}}=m_{C_{t}}^{ul}$, and the excess sample numbers are evenly allocated to $C_{s}$ blocks. $m_{C_{s}}$ should be updated as

(25) $$m_{C_{s}}=round\left(\frac{S-N_{t}\cdot m_{C_{t}}^{ul}}{N_{s}}\right).$$

For complex images, there are three categories, $C_{s}$, $C_{t_{1}}$, and $C_{t_{2}}$. A fixed initial sampling number is assigned to $C_{s}$, $m_{C_{s}}=m_{l}$, and the remaining sampling number is evenly allocated to $C_{t_{1}}$ and $C_{t_{2}}$. Then, a sampling number reducing is taken for $C_{t_{1}}$ blocks, and the saved sampling number is given to the $C_{t_{2}}$ blocks,

(26) $$m_{C_{t_{1}}}=round\left(\frac{S-m_{l}\cdot N_{s}}{N_{t_{1}}+N_{t_{2}}}-\beta \right),$$

and

(27) $$m_{C_{t_{2}}}=round\left(\frac{S-m_{l}\cdot N_{s}}{N_{t_{1}}+N_{t_{2}}}+\beta \frac{N_{t_{1}}}{N_{t_{2}}}\right),$$

where $m_{C_{t_{1}}}$ and $m_{C_{t_{2}}}$ are the sampling numbers of the $C_{t_{1}}$ and $C_{t_{2}}$ blocks, $N_{t_{1}}$ and $N_{t_{2}}$ are the numbers of $C_{t_{1}}$ and $C_{t_{2}}$ blocks, and β is the parameter that controls the reducing number. It is also necessary to check if $m_{C_{t_{1}}}$ is too small or $m_{C_{t_{2}}}$ is too big. In this paper, $m_{C_{t_{1}}}$ is limited to be no less than 1.04 times the size of $m_{C_{s}}$, and $m_{C_{t_{2}}}$ is limited to be no greater than $m_{C_{t}}^{ul}$. This can be guaranteed by adjusting the value of β.

Finally, when the final sampling number m for each block is decided, the supplementary sampling operation should be executed. For each block, if $m>m_{l}$, a new supplementary matrix $\Phi_{s}\in R^{m_{s}\times n}$ is generated, and the supplementary measurement $y_{i}^{s}\in R^{m_{s}}$ can be obtained, where $m_{s}=m-m_{l}$. Then, the final CS measurement $y_{i}$ can be obtain by concatenating $y_{i}^{l}$ and $y_{i}^{s}$.

### 3.4. Reconstruction

This work mainly focuses on the adaptive sampling rate method. When the sampling rate is reasonably allocated at the sampling end, the commonly used reconstruction methods can obtain good results. A classic reconstruction method, SPGL1 [31,32], was selected to reconstruct signal in this method. Using the sensing dictionary A, measurement $y_{i}$, and measurement matrix $\Phi_{b}$, the approximate sparse solution ${\tilde{a}}_{i}$ can be solved:

(28) $$\underset{{\tilde{a}}_{i}\in R^{N}}{{\tilde{a}}_{i}=min{\left\|{\tilde{a}}_{i}\right\|}_{1}}\;such\;that\;{\left\|A{\tilde{a}}_{i}-y_{i}\right\|}_{2}\leq \epsilon .$$

The approximate solution ${\tilde{x}}_{i}$ of an image block is

(29) $${\tilde{x}}_{i}=D{\tilde{a}}_{i},\;i\in 1,2,3,\ldots ,I.$$

## 4. Experiments

### 4.1. Parameter Settings

The proposed method was validated on a set of standard test images, which are accessible via the link https://github.com/eclipsetb/academic/blob/main/testImages.zip (accessed on 23 June 2025).

To verify the method’s stability, the method was tested on both 512×512 high- resolution images and 256×256 low-resolution images. Each image was partitioned into 16 × 16 blocks, and individual tests are performed on each image with total sampling rates of 0.1, 0.2, 0.3, 0.4, and 0.5.

The parameters used in the block classification operation are listed in Table 2. Since $K_{s}$ controls the number of iterations of the Algorithm 1, the smaller it is, the faster the program runs. The actual tests showed that $K_{s}$ can be set to 1 to distinguish smooth blocks while obtaining the fastest running speed. $\tau_{s}$ is a normalized threshold that is used to distinguish the smooth blocks. $K_{t}$ and $\tau_{t}$ are the similar parameters; however, they are used to distinguish between the $C_{t_{1}}$ blocks and $C_{t_{2}}$ blocks.

The sampling rate allocation parameters are shown in Table 3. These parameters determine the final sampling number, and they are not related to the block classification method.

### 4.2. Simulation Results

In this section, the proposed method is compared with the ABCS-SD [7] ABCS-MC [10], ABCS-Entropy [8], and BCS [15] methods. Among them, the ABCS-SD, ABCS-MC, and ABCS-Entropy methods are adaptive sampling rate methods, and the BCS is a traditional fixed-rate method. The ABCS-SD and BCS methods were not optimized for the reconstruction method and were reconstructed using the SPGL1 algorithm, consistent with the proposed method. For the other methods with optimized reconstruction processes, their optimized processes were used in the experiments.

**Figure 4:**
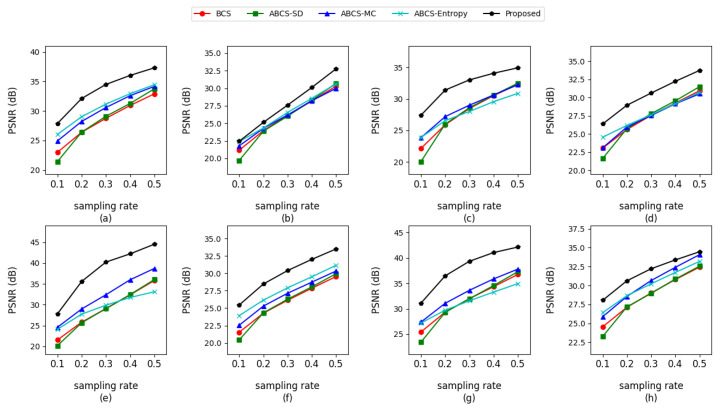
PSNR comparison of various reconstruction algorithms for different sampling rates with 512 × 512 images. (**a**) Lena; (**b**) Barbara; (**c**) Peppers; (**d**) Goldhill; (**e**) Cameraman; (**f**) Pirate; (**g**) Luna; (**h**) Heron.

The PSNRs (peak signal-to-noise ratios) of the reconstructed high-resolution images for different sampling rates are shown in Table 4. The reconstruction quality plots are illustrated in Figure 4. As shown in Table 4 and Figure 4, the method proposed in this article achieved the best PSNRs for all test images. We considered that this was mainly due to the proposed method being able to accurately identify smooth and texture blocks as well as the better sparse representation ability of the overcomplete dictionary. We also would like to mention that Figure 4 shows that when the sampling rate increases from the lowest values, such as increasing from 0.1 to 0.2 or 0.3, the reconstruction quality of the proposed method improves faster than that of the other methods. When the sampling rate is sufficient, such as increasing from 0.4 to 0.5, the quality improvement provided by the proposed method slows. It can be considered that when the sampling rate is insufficient, the proposed method quickly allocates the increased sampling resources to the complex blocks that need these resources. It also shows that this method relatively accurately identifies texture blocks.

**Figure 5:**
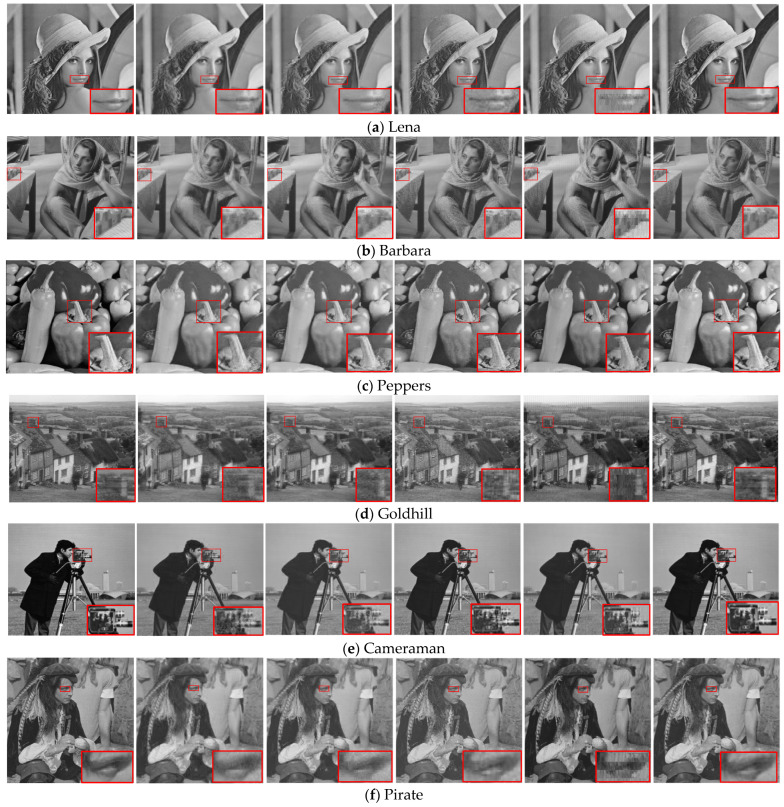

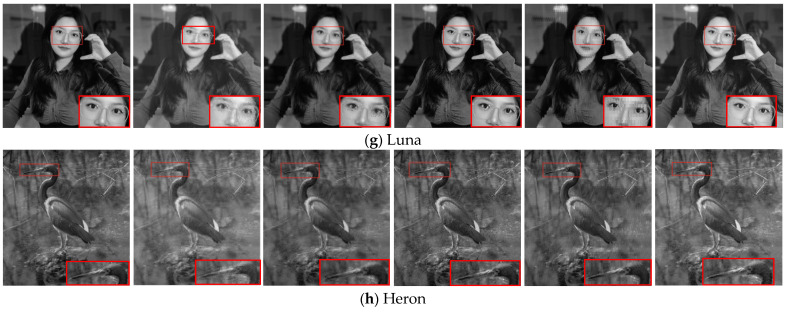
Visual quality comparison of various reconstruction algorithms on 512 × 512 images at a sampling rate r=0.3. The images from left to right in each row are the original image and those reconstructed with the BCS, ABCS-SD, ABCS-MC, ABCS-Entropy, proposed methods.

Figure 5 shows the visual effects of the different methods in reconstructing high-resolution images when the total sampling rate r=0.3. Overall, the proposed method has better visual effects with all test images. For more complex parts of these images, the proposed method often achieves better reconstruction quality than the other methods. Simultaneously, a relatively consistent reconstruction quality can be achieved for both the texture blocks and the smooth blocks, and the blocking effect is not obvious. This can be illustrated from another aspect: the proposed method can identify smooth blocks and texture blocks well and assign them reasonable sampling rates.

The method was also tested on some low-resolution images. And the PSNR results are shown in Table 5. It can be seen that the proposed method also achieves the best PSNRs in these low-resolution images.

Considering that the proposed method was designed for distributed applications, to evaluate the complexity of the sampling operations, the running time was also tested using an embedded device. A Raspberry Pi 5 was used as the testing device, its CPU was an Arm Cortex-A76(2.4 GHz), and it had 4GB RAM. Its energy consumption was less than 20 Watts, and its current price was less than USD 60.

**Figure 6:**
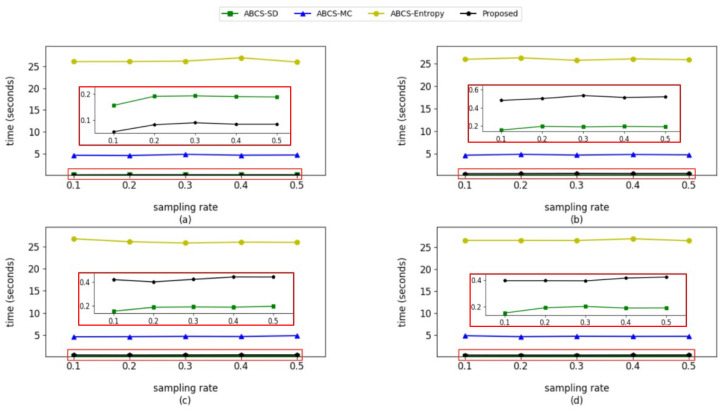
Computation times for various adaptive algorithms at different sampling rates. A Raspberry Pi 5 was used as the test device. The large red rectangle contains an enlarged display of the small red rectangle. The tested images were (**a**) Cameraman (simple image); (**b**) Barbara (complex images); (**c**) Peppers (complex images); (**d**) Lena (complex images).

Each method was tested 10 times on the device, and the average values are shown in Figure 6.

It can be seen from Figure 6 that, when the test image is simple, our method outperforms all other methods in speed, and when the test image is more complex, the proposed method is slightly slower than the ABCS-SD method, but the gap is not large. The experimental results show that the proposed method is a low-computation-complexity method.

## 5. Summary

This paper proposes an innovative CS method based on an overcomplete ridgelet dictionary. This method can estimate the sparsity of image blocks based on CS measurements, without dependency on the original image. At the same time, the method is simple as well as accurate and adaptively allocate reasonable sampling rates for blocks, achieving better reconstruction quality at a given sampling rate. These characteristics make it particularly suitable for resource-constrained systems, such as monitoring systems, sensor network systems, or satellite systems. The experimental results show the effectiveness of the proposed method. Compared with the existing methods, images with a higher PSNR and better visual effects can be reconstructed with a similar or faster running time.

In our future work, we will explore two research directions. One is to further study the specific performance of this method in practical application scenarios, involving testing and optimizing its performance on actual scene images rather than standard test images. Second, noting that the core mechanism of this method involves classifying specific signals and considering the demonstrated ability of deep learning methods on classification tasks, we plan to explore applications of deep learning methods in ARCS.

## Figures and Tables

**Table 1:** Comparison of existing methods.

Method	Original Image Dependence	Computational Cost	Reconstruction Quality
ABCS-RW [4]	Yes	Medium	High
STD-BCS-SPL [5]	Yes	Low	Low
ABCS-ARS [6]	Yes	Medium	Medium
ABCS-SD [7]	Yes	Low	Medium
ABCS-Entropy [8]	No	High	Medium
ABCS-MC [10]	No	Medium	Medium
Zigzag EMD [11]	No	High	Medium
ABCS-IRD [12]	No	Medium	Medium

**Table 2:** Block classification parameters.

$K_{s}$	$\tau_{s}$	$K_{t}$	$\tau_{t}$
1	0.1	4	0.01

**Table 3:** Rate allocation parameters.

$m_{l}$	$m_{C_{t}}^{ul}$	β
23	240	15

**Table 4:** Comparison of PSNRs (dB) at different sampling rates for 512 × 512 images.

Images	Methods	Sampling Rates				
		0.1	0.2	0.3	0.4	0.5
Lena	BCS	23.00	26.40	28.75	30.93	32.90
	ABCS-SD	21.43	26.42	29.10	31.27	33.75
	ABCS-MC	24.93	28.23	30.58	32.59	34.16
	ABCS-Entropy	26.04	29.03	31.16	32.93	34.45
	Proposed	**27.88**	**32.12**	**34.49**	**36.02**	**37.30**
Barbara	BCS	21.16	23.95	26.14	28.18	30.31
	ABCS-SD	19.70	23.88	26.02	28.29	30.72
	ABCS-MC	21.78	24.28	26.19	28.23	29.99
	ABCS-Entropy	22.39	24.33	26.56	28.57	30.64
	Proposed	**22.43**	**25.14**	**27.60**	**30.12**	**32.78**
Peppers	BCS	22.13	25.89	28.47	30.51	32.32
	ABCS-SD	20.04	25.99	28.59	30.59	32.48
	ABCS-MC	23.84	27.18	29.00	30.62	32.24
	ABCS-Entropy	23.87	26.55	28.00	29.56	30.87
	Proposed	**27.40**	**31.40**	**33.01**	**34.07**	**34.93**
Goldhill	BCS	23.07	25.61	27.56	29.25	30.94
	ABCS-SD	21.64	25.74	27.79	29.56	31.50
	ABCS-MC	23.10	25.96	27.54	29.14	30.52
	ABCS-Entropy	24.56	26.18	27.64	29.17	30.73
	Proposed	**26.38**	**28.95**	**30.62**	**32.23**	**33.73**
Cameraman	BCS	21.55	25.83	29.07	32.37	35.82
	ABCS-SD	20.19	25.68	29.07	32.44	36.04
	ABCS-MC	24.60	28.99	32.40	35.99	38.71
	ABCS-Entropy	24.12	27.76	29.88	31.74	33.12
	Proposed	**27.82**	**35.59**	**40.23**	**42.21**	**44.52**
Pirate	BCS	21.53	24.28	26.14	27.87	29.56
	ABCS-SD	20.43	24.33	26.29	28.07	29.96
	ABCS-MC	22.54	25.30	27.15	28.75	30.35
	ABCS-Entropy	23.93	26.16	27.93	29.52	31.17
	Proposed	**25.45**	**28.48**	**30.42**	**32.03**	**33.53**
Luna	BCS	25.42	29.33	31.98	34.35	36.75
	ABCS-SD	23.42	29.28	31.97	34.56	37.30
	ABCS-MC	27.37	31.07	33.63	35.87	37.80
	ABCS-Entropy	27.19	29.71	31.57	33.28	34.95
	Proposed	**31.10**	**36.47**	**39.34**	**41.06**	**42.14**
Heron	BCS	24.55	27.13	29.02	30.79	32.44
	ABCS-SD	23.27	27.18	28.97	30.86	32.57
	ABCS-MC	25.88	28.56	30.66	32.41	34.09
	ABCS-Entropy	26.42	28.66	30.23	31.74	33.20
	Proposed	**28.05**	**30.63**	**32.22**	**33.39**	**34.47**

**Table 5:** Comparison of PSNRs (dB) at different sampling rates on 256 × 256 images.

Images	Methods	Sampling Rates				
		0.1	0.2	0.3	0.4	0.5
Lena	BCS	20.19	23.58	25.89	27.83	29.90
	ABCS-SD	18.92	23.19	25.85	28.12	30.80
	ABCS-MC	22.32	25.73	28.13	30.25	32.21
	ABCS-Entropy	23.55	26.25	28.40	30.30	32.23
	Proposed	**25.01**	**28.63**	**31.00**	**33.61**	**35.41**
Heron	BCS	23.20	25.45	27.42	29.30	31.17
	ABCS-SD	22.30	25.54	27.43	29.35	31.28
	ABCS-MC	24.31	27.17	29.25	31.33	33.38
	ABCS-Entropy	25.08	27.01	28.63	30.12	31.49
	Proposed	**26.68**	**29.60**	**31.79**	**33.55**	**35.31**
Goldhill	BCS	21.26	24.00	26.18	28.24	30.14
	ABCS-SD	20.29	24.51	26.42	28.47	30.66
	ABCS-MC	22.18	25.06	27.02	28.68	30.07
	ABCS-Entropy	23.38	24.92	26.24	27.69	29.21
	Proposed	**25.24**	**28.07**	**30.19**	**32.17**	**34.06**

## Data Availability

Data are contained within this article.

## References

[1] Masoum A., Meratnia N., Havinga P.J.M. Compressive Sensing Based Data Collection in Wireless Sensor Networks. Proceedings of the 2017 IEEE International Conference on Multisensor Fusion and Integration for Intelligent Systems (MFI).

[2] Hsieh S.-H., Liang W.-J., Lu C.-S., Pei S.-C. (2020). Distributed Compressive Sensing: Performance Analysis with Diverse Signal Ensembles. IEEE Trans. Signal Process..

[3] Lin W., Dong L. (2006). Adaptive Downsampling to Improve Image Compression at Low Bit Rates. IEEE Trans. Image Process..

[4] Zhu S., Zeng B., Gabbouj M. Adaptive Reweighted Compressed Sensing for Image Compression. Proceedings of the 2014 IEEE International Symposium on Circuits and Systems (ISCAS).

[5] Zhang J., Xiang Q., Yin Y., Chen C., Luo X. (2017). Adaptive Compressed Sensing for Wireless Image Sensor Networks. Multimed. Tools Appl..

[6] Monika R., Dhanalakshmi S. (2024). An Optimal Adaptive Reweighted Sampling-Based Adaptive Block Compressed Sensing for Underwater Image Compression. Vis. Comput..

[7] Yu Y., Wang B., Zhang L. (2010). Saliency-Based Compressive Sampling for Image Signals. IEEE Signal Process. Lett..

[8] Li R., Duan X., He W., You L. (2020). Entropy-Assisted Adaptive Compressive Sensing for Energy-Efficient Visual Sensors. Multimed. Tools Appl..

[9] Itti L., Koch C., Niebur E. (1998). A Model of Saliency-Based Visual Attention for Rapid Scene Analysis. IEEE Trans. Pattern Anal. Mach. Intell..

[10] Li R., He W., Liu Z., Li Y., Fu Z. (2018). Saliency-Based Adaptive Compressive Sampling of Images Using Measurement Contrast. Multimed. Tools Appl..

[11] Wang W., Chen J., Zhang Y., Xia J., Zeng X. (2023). Adaptive Compressed Sampling Based on EMD for Wireless Sensor Networks. IEEE Sens. J..

[12] Wang W., Jin X., Quan D., Zhu M., Wang X., Zheng M., Li J., Chen J. (2024). Rate Adaptive Compressed Sampling Based on Region Division for Wireless Sensor Networks. Sci. Rep..

[13] Song Z., Chen J. (2025). Adaptive Rate Compression for Distributed Video Sensing in Wireless Visual Sensor Networks. Vis. Comput..

[14] Unde A.S., Pattathil D.P. (2020). Adaptive Compressive Video Coding for Embedded Camera Sensors: Compressed Domain Motion and Measurements Estimation. IEEE Trans. Mob. Comput..

[15] Gan L. Block Compressed Sensing of Natural Images. Proceedings of the 2007 15th International Conference on Digital Signal Processing.

[16] Olshausen B.A., Field D.J. (1997). Sparse Coding with an Overcomplete Basis Set: A Strategy Employed by V1?. Vision Res..

[17] Olshausen B.A., Field D.J. (1996). Emergence of Simple-Cell Receptive Field Properties by Learning a Sparse Code for Natural Images. Nature.

[18] Figueras i Ventura R.M., Vandergheynst P., Frossard P. (2006). Low-Rate and Flexible Image Coding with Redundant Representations. IEEE Trans. Image Process..

[19] Donoho D.L., Elad M. (2003). Optimally Sparse Representation in General (Nonorthogonal) Dictionaries via ℓ^1^ Minimization. Proc. Natl. Acad. Sci. USA.

[20] Randall P.A. (2009). Sparse Recovery via Convex Optimization.

[21] Candès J.E., Eldar C.Y., Needel D., Randall P. (2011). Compressed Sensing with Coherent and Redundant Dictionaries. Appl. Comput. Harmon. Anal..

[22] Jin S., Sun W., Huang L. (2023). Joint Optimization Methods for Gaussian Random Measurement Matrix Based on Column Coherence in Compressed Sensing. Signal Process..

[23] Yang C., Pan P., Ding Q. (2022). Image Encryption Scheme Based on Mixed Chaotic Bernoulli Measurement Matrix Block Compressive Sensing. Entropy.

[24] Tausiesakul B. Basis Pursuit and Linear Programming Equivalence: A Performance Comparison in Sparse Signal Recovery. Proceedings of the 2022 7th International Conference on Smart and Sustainable Technologies (SpliTech).

[25] Figueiredo M.A.T., Nowak R.D., Wright S.J. (2007). Gradient Projection for Sparse Reconstruction: Application to Compressed Sensing and Other Inverse Problems. IEEE J. Sel. Top. Signal Process..

[26] Lin L., Liu F., Jiao L., Yang S., Hao H. (2018). The Overcomplete Dictionary-Based Directional Estimation Model and Nonconvex Reconstruction Methods. IEEE Trans. Cybern..

[27] Lin L., Liu F., Jiao L. (2014). Compressed Sensing by Collaborative Reconstruction on Overcomplete Dictionary. Signal Process..

[28] Begovic B. (2014). Dictionary Learning for Scalable Sparse Image Representation. Adv. Signal Process..

[29] Pati Y.C., Rezaiifar R., Krishnaprasad P.S. Orthogonal Matching Pursuit: Recursive Function Approximation with Applications to Wavelet Decomposition. Proceedings of the Proceedings of 27th Asilomar Conference on Signals, Systems and Computers.

[30] Singh D., Singh B. (2020). Investigating the Impact of Data Normalization on Classification Performance. Appl. Soft Comput..

[31] Van Den Berg E., Friedlander M.P. (2009). Probing the Pareto Frontier for Basis Pursuit Solutions. SIAM J. Sci. Comput..

[32] Van Den Berg E., Friedlander M.P. (2011). Sparse Optimization with Least-Squares Constraints. SIAM J. Optim..

